# Spirituality in palliative care

**DOI:** 10.1186/s12904-022-01116-x

**Published:** 2023-01-04

**Authors:** Barry Quinn, Michael Connolly

**Affiliations:** 1 School of Nursing and Midwifery, Queen’s University Belfast, Belfast, Northern Ireland; 2 UCD School of Nursing, Midwifery and Health Systems, University College Dublin, Dublin, Ireland

## Abstract

Although being recognized by the World Health Organization as an essential domain of palliative care, spiritual care is still one of the most neglected component of the healthcare system. In this editorial, we set the context and invite contributions for a *BMC Palliative Care* Collection of articles titled ‘Spirituality in Palliative Care’.

Ben Okri, the great novelist, wrote about the importance of sharing stories, a process which he described as enabling a greater connection among people to the common bond of ‘human destiny, human suffering, and human transcendence’ [1]. The stories that Okri alludes to, are very much present and numerous within the field of advanced diseases and palliative care. Central to these stories, shared and heard, are the beliefs, values and the personal meanings about life that makes each person unique, alongside the common bond with others and with the world in which they live.

Interestingly, Okri’s views closely reflect the definition of spirituality provided by the Spirituality Reference Group of the European Association of Palliative Care (EAPC), which states that ‘spirituality is the dimension of human life that relates to the way people/community experience, express and/or seek meaning, purpose and transcendence, and the way they connect to the moment, to self, to others, to nature, to the significant and/or the sacred’ [2].

Although spirituality is a theoretical concept and its broadness and uniqueness make it difficult to be defined, many authors have previously addressed the spiritual dimension of humanity and very vividly described it as the deepest part of who we are, the concern with ultimate questions, the need to find meaning, purpose and fulfilment in life, suffering and death [3, 4]. When someone receives a life-threatening prognosis, this spiritual process of exploring meaning and purpose inevitably takes on a new urgency, which may imply the need to address personal beliefs and values that have been disrupted and disturbed by the life events [5].

Therefore, it is evident that life threatening or life changing events are not just characterized by physical symptoms. Patients at the end-of-life may suffer spiritual distress, which can include deep inner questioning and loss of meaning and purpose in life. Indeed, as stated by Viktor Frankl, ‘man is not destroyed by suffering: he is destroyed by suffering without meaning’ [6]. This spiritual distress has also been well described in a recent study, where people living with advanced cancer reported having hidden pain and suffering—not recognized by their family or the healthcare team—which left them feeling alone and isolated [7].

It is then becoming clear that a healthcare approach that mainly focuses on alleviating physical pain is no longer adequate and that responding to people with palliative care needs inevitably requires engaging with the spiritual dimension of each person. Amidst the uncertainty and the painful realities associated to life threatening conditions, people with advanced disease openly express the importance of having their spiritual necessities acknowledged and addressed by healthcare professionals. This may just imply a simple act of kindness with a caring attitude, a compassionate presence, a warm and empathic connection [8]. Growing evidence has also supported the benefits of providing spiritual care at the end of life on clinical outcomes, highlighting the importance of its integration in palliative care interventions [9].

While we have come a long way in easing physical discomfort and improving physical wellbeing of patients at the end-stage of life, spiritual care is still the most neglected component of palliative care [10]. This reluctance may stem either from a narrow and prejudiced view of what spirituality might mean or from the adoption of healthcare models focused on just ‘fix’ and ‘solve’. However, following the recognised positive impact of spiritual care on patients’ and carers’ wellbeing, initiatives have been established in the last decade to promote the integration of this dimension of care in research and clinical settings. In 2010, the European Association for Palliative Care (EAPC) founded a Spiritual Care Taskforce, which aims to ‘further evidence-based spiritual care by developing an agenda to inform research in this area, to improve staff competence and confidence and outcomes for patients and carers’. The Global Network for Spirituality and Health (GNSAH) was formed in the USA in 2013, with one of the explicit goals being to build ‘the knowledge and evidence base related to spirituality and health’.

In alignment with the World Health Organisation (WHO) statement that it is the ethical duty of healthcare professionals to alleviate not only physical pain but also spiritual suffering [11], we are now welcoming submissions to our new Collection of articles titled ‘Spirituality in Palliative Care’. More details can be found here: https://www.biomedcentral.com/collections/SPPC. This is a real opportunity to engage with this core dimension of palliative care, by contributing with original articles focusing on the challenges that exist in assessing spiritual care needs and providing appropriate spiritual interventions around the world. We warmly invite authors to submit their works reporting new multi-disciplinary approaches to promote the integration of spiritual support in the hospital, hospice and home settings. Studies on novel outcome measurements that appropriately capture the effects of the variety of spiritual care interventions are also welcome. As spirituality is something that is unique and personal, we would also like to receive reports on how spiritual needs differ among individuals of various ages, cultures and genders.

We hope that this Collection will provide a useful platform for shared learning for all healthcare professionals working in the field of palliative care, who play a vital role in responding to the spiritual needs of those they support.

## Data Availability

Not applicable.

## Abbreviations

EAPC: European Association of Palliative Care
GNSAH: Global Network for Spirituality and Health
WHO: World Health Organisation
